# The relationship between emotional competence, psychological resilience, and job burnout among primary school physical education teachers: the mediating role of psychological resilience

**DOI:** 10.3389/fpsyg.2026.1857739

**Published:** 2026-08-06

**Authors:** Xunbin Li, Heng Liu

**Affiliations:** 1 School of Sports and Leisure, Sichuan Tourism University, Chengdu, China; 2 College of Physical Education, Chongqing University, Chongqing, China

**Keywords:** emotional competence, job burnout, mediating mechanism, primary school physical education teachers, psychological resilience

## Abstract

**Objective:**

This study aimed to explore the relationships between emotional competence, psychological resilience and job burnout among primary school physical education (PE) teachers, and examine the mediating role of psychological resilience.

**Methods:**

A total of 228 teachers from 24 primary schools in Chengdu were recruited via stratified random sampling. All participants completed three validated scales measuring emotional competence, psychological resilience and job burnout.

**Results:**

Both emotional competence and psychological resilience were significantly and negatively associated with job burnout among primary school PE teachers (*β* = −0.49, *p* < 0.001; *β* = −0.52, *p* < 0.001). Among the dimensions of emotional competence, emotion regulation showed the strongest correlation with job burnout (*r* = −0.64, *p* < 0.01); among the dimensions of psychological resilience, resilience showed the highest correlation with job burnout (*r* = −0.61, *p* < 0.01). Psychological resilience played a partial mediating role in the association between emotional competence and job burnout, accounting for 55.1% of the total effect.

**Conclusion:**

Emotional competence of primary school PE teachers is directly associated with job burnout and also indirectly related to burnout levels via psychological resilience. It is suggested that education administrators improve primary school PE teachers’ emotion regulation skills and organize systematic psychological resilience training to reduce job burnout.

## Introduction

With China’s ongoing emphasis on the physical and mental health of students in basic education, the importance of primary school physical education curricula has become increasingly prominent. Physical education (PE) teachers, as key agents in curriculum implementation, have seen their mental health status and professional conditions gradually become important topics in educational research and policy formulation (Fernández et al., 2024). Especially against the backdrop of the comprehensive implementation of China’s “double reduction” policy (i.e., reducing homework burden and off-campus tutoring for students in compulsory education) (Chen and Lin, 2026), school physical education has been endowed with more educational functions. PE teachers are not only responsible for teaching tasks but also need to organize extracurricular activities, plan school sports events, and guide students in competitions. Their work is fast-paced, task-heavy, and involves multiple roles, making them highly susceptible to emotional exhaustion and job burnout (Chen et al., 2025).

Job burnout (Tian and Cheng, 2025; Yu and Cheng, 2024) refers to a three-dimensional psychological syndrome—characterized by emotional exhaustion, depersonalization, and reduced personal accomplishment—that occurs under long-term work stress. As a group engaged in high emotional labor, teachers’ job burnout not only affects their individual physical and mental health but may also indirectly impact students’ learning motivation, emotion regulation ability, and physical and mental development through teaching behaviors, classroom atmosphere, and teacher-student interactions (Madigan and Kim, 2021). Compared to teachers of other subjects, primary school PE teachers operate in more open teaching environments, face greater challenges in classroom management, higher risks to student safety, and often encounter identity dilemmas as a “marginal subject,” leading to job burnout issues that are more concealed yet severe (Alsalhe et al., 2021).

As a typical occupational psychological problem caused by long-term work stress, job burnout is closely bound up with individual emotional management ability. Teachers’ daily work requires continuous emotional labor, and the level of emotional competence directly affects their emotional state and stress coping ability, and further influences the occurrence and development of job burnout. On this basis, we further clarify the connotation and dimensions of emotional competence. Emotional competence refers to an individual’s comprehensive ability to identify, understand, regulate, and utilize emotions (Mehler et al., 2024). Teachers with high emotional competence are more effective in managing classroom conflicts, maintaining positive emotional states, and enhancing their sense of teaching efficacy and professional wellbeing (Jennings and Greenberg, 2009). In the context of physical education, teachers frequently need to respond to unexpected situations such as student behavior fluctuations, classroom disorder, parental misunderstandings, and complaints. Emotion regulation ability becomes key to maintaining teaching order and teacher-student relationships (Cheng and Liu, 2024). Particularly, emotion regulation is considered the core dimension of teachers’ emotional labor, directly affecting their level of emotional exhaustion (Aldrup et al., 2024). However, current research on the mechanism linking emotional competence to job burnout has mostly focused on service-oriented professions such as kindergarten teachers (Zhang et al., 2025) and nurses (Guillouët et al., 2025), resulting in relatively limited empirical studies on primary school PE teachers.

On the other hand, psychological resilience, as the ability to recover and adapt in the face of stress, setbacks, or adversity, is considered an important protective factor for alleviating job burnout (Durmuş et al., 2024). Psychological resilience can not only directly alleviate emotional exhaustion and depersonalization but also enhance an individual’s sense of professional control and accomplishment by strengthening positive emotional experiences and coping strategies (Liu et al., 2024). Among teacher populations, psychological resilience can indirectly reduce burnout levels by regulating work stress and enhancing the perception of social support (Li, 2023). However, whether psychological resilience mediates this relationship has not been systematically examined specifically among primary school PE teachers, and the specific pathways remain to be empirically validated.

Accordingly, this study selects primary school PE teachers as research subjects and constructs a mediation model to examine how emotional competence affects job burnout via psychological resilience. It aims to reveal the internal mechanism between emotional competence, psychological resilience, and job burnout among primary school PE teachers from the perspective of positive psychology, enrich empirical research in the field of teacher mental health, and provide a theoretical basis and practical reference for educational administrators to develop targeted psychological support policies and intervention programs. This study offers two notable contributions. First, it examines the mediating role of psychological resilience in the link between emotional competence and job burnout among primary school PE teachers, a relationship not previously explored in this specific occupational group. Second, it adopts a positive psychology perspective focusing on protective resources, providing empirical evidence to inform teacher mental health training and school-based intervention programs. Research hypotheses: H1: Emotional competence of primary school PE teachers significantly negatively predicts job burnout; H2: Psychological resilience of primary school PE teachers significantly negatively predicts job burnout; H3: Psychological resilience plays a partial mediating role in the association between emotional competence and job burnout.

## Materials and methods

### Participants

This study was approved by the Ethics Committee of Sichuan Sports College (No. 2025020) and conforms to the Declaration of Helsinki. This study adopted stratified random sampling to recruit participants from six administrative districts of Chengdu. Inclusion criteria: Full-time primary school PE teachers; Teaching experience of at least 1 year; Voluntary participation and willingness to sign the electronic informed consent form; Exclusion criteria: Part-time teachers or those without a PE professional background; Those who had received psychological treatment in the past 6 months; Questionnaires with incomplete or patterned responses were excluded to guarantee data quality. The above inclusion and exclusion criteria ensured that all participants were professional on-the-job primary school PE teachers with stable working experience and normal mental status, which guaranteed the validity of data for exploring occupational burnout among PE teachers.

First, we took administrative districts as the primary sampling layer and randomly selected 6 schools from each district, with a total of 24 schools included; subsequently, researchers contacted the district education bureaus and school principals, explained the research purpose and ethical safeguards, and obtained formal permission. From the list of full-time, officially appointed PE teachers provided by each school’s PE teaching research group, 10 teachers were randomly selected. Then, a QR code link was generated via the Questionnaire Star platform, and teachers independently filled out the questionnaire by scanning the code with their mobile phones during a unified time period. The system included logical skip patterns and consistency checks. The average completion time was 5–10 min. During the data cleaning phase, questionnaires with completion times less than 1 min, 10 consecutive questions with the same option, missing values exceeding 10%, and obviously patterned responses were excluded. A total of 240 teachers accessed the online questionnaire via QR code. After excluding 12 invalid responses, 228 valid questionnaires were retained, yielding an effective response rate of 95.0%. The sample was predominantly male (156 individuals, 68.4%), age was mainly 31–40 years old (42.6%), teaching experience was mostly 6–15 years (44.7%), educational attainment was 100% bachelor’s degree or above, with master’s degree accounting for 13.2%, indicating good representativeness (Table 1). Post-hoc power analysis was conducted using G*Power 3.1. For linear regression with *f*^2^ = 0.15 (medium effect), *α* = 0.05, and 2 predictors, the achieved power was 0.98, exceeding the conventional 0.80 threshold, confirming adequate sample size.

**Table 1:** Participant information.

Item	Classification	Number of people (*n* = 228)	Percentage (%)
Gender	Male	156	68.4
	Female	72	31.6
Age	≤30 years	89	39.0
	31–40 years	97	42.6
	≥41 years	42	18.4
Teaching experience	1–5 years	78	34.2
	6–15 years	102	44.7
	≥16 years	48	21.1
Education	Bachelor’s degree	198	86.8
	Master’s degree or above	30	13.2

### Scale measurements

Schutte Emotional Intelligence Scale (SEIS). Based on the scale developed by Jonker and Vosloo (2008), revised by Xu et al. (2019) for the Chinese population. It consists of four dimensions: Emotional perception (9 items), Emotion regulation (10 items), Emotional understanding (8 items), and Emotional utilization (6 items). Scoring method: A 5-point Likert scale (1 = Strongly Disagree, 5 = Strongly Agree). In this study, Cronbach’s *α* coefficient was 0.89, and the Cronbach’s *α* coefficients for each dimension ranged from 0.78 to 0.85, indicating good reliability.

Connor–Davidson Resilience Scale (CD-RISC). Based on the scale developed by Connor and Davidson (2023), revised by Ye et al. (2017) for the Chinese population. It consists of three dimensions: Resilience (13 items), Strength (8 items), and Optimism (4 items). Scoring method: Likert 4-point scale (0 = Never, 4 = Almost Always). In this study, Cronbach’s *α* = 0.87, with dimension *α* values between 0.80 and 0.86, indicating good reliability.

Maslach Burnout Inventory-Educators Survey (MBI-ES). Based on the scale developed by Kokkinos (2006), revised by Zhang (2022) for the Chinese population, used with formal permission from the author. It consists of three dimensions: Emotional exhaustion (5 items), Depersonalization (5 items), and Reduced personal accomplishment (6 items). Scoring method: Likert 5-point scale (1 = Never, 5 = Daily). In this study, Cronbach’s *α* = 0.91, with dimension *α* values between 0.84 and 0.89, indicating good reliability.

### Statistical analysis

Data analysis was performed using SPSS 26.0 and PROCESS v3.5. First, descriptive statistics and Pearson correlation analysis were conducted to understand the overall levels of emotional competence, psychological resilience, job burnout, and the relationships between variables. Then, multiple linear regression was used to test predictive effects, followed by multicollinearity diagnosis. The Shapiro–Wilk test was used to verify the normal distribution of residuals, testing the independent predictive effects of emotional competence and psychological resilience on job burnout. Finally, the researchers used Hayes’ Model 4 to test the mediating effect of psychological resilience in the path through which emotional competence affects job burnout. This analysis controlled for demographic variables (e.g., gender, age, and teaching experience) and used 5,000 Bootstrap samples. All regression models underwent multicollinearity diagnosis, and residuals were approximately normally distributed, meeting the prerequisites for linear regression (Yu and Cheng, 2024). The Benjamini-Hochberg False Discovery Rate (FDR) procedure was applied to correct for multiple comparisons; all reported significant results survived correction. All regression and mediation results reported both unstandardized coefficients (*B*) with standard errors and standardized coefficients (*β*) for reference. Significance level *α* = 0.05.

## Results

Results in Table 2 show that the total average score of emotional competence among participants was 3.22 ± 0.83. Among its dimensions, the score for “emotion regulation” was the highest (3.41 ± 0.89), while that for “emotional utilization” was the lowest (3.05 ± 0.80). The total average score for psychological resilience was 3.24 ± 0.49, with “Resilience” scoring the highest (3.35 ± 0.61) and “Optimism” scoring the lowest (3.18 ± 0.52). The total average score for job burnout was 2.52 ± 0.45, below the theoretical median of 3. Among the dimensions, “reduced personal accomplishment” scored the highest (2.60 ± 0.58), and “Depersonalization” scored the lowest (2.45 ± 0.51). Table 3 shows that participants’ emotional competence was significantly negatively correlated with job burnout (*r* = −0.66, *p* < 0.01), with “Emotion regulation” showing the strongest correlation with burnout (*r* = −0.64). Psychological resilience was also significantly negatively correlated with job burnout (*r* = −0.70, *p* < 0.01), with “Resilience” showing the strongest correlation with burnout (*r* = −0.61). Emotional competence was significantly positively correlated with psychological resilience (*r* = 0.68, *p* < 0.01).

**Table 2:** Descriptive statistics of variables (*n* = 228).

Variable	Dimension	Mean ± Standard deviation (scores)
Emotional competence	Emotional perception	3.12 ± 0.78
	Emotion regulation	3.41 ± 0.89
	Emotional understanding	3.28 ± 0.85
	Emotional utilization	3.05 ± 0.80
	Total score	3.22 ± 0.83
Psychological resilience	Resilience	3.35 ± 0.61
	Strength	3.20 ± 0.55
	Optimism	3.18 ± 0.52
	Total score	3.24 ± 0.49
Job burnout	Emotional exhaustion	2.52 ± 0.48
	Depersonalization	2.45 ± 0.51
	Reduced personal accomplishment	2.60 ± 0.58
	Total score	2.52 ± 0.45

**Table 3:** Correlation analysis of emotional competence, psychological resilience, and job burnout (*r* values, *n* = 228).

Variable	1	2	3	4	5	6
1. Emotional competence	–					
2. Emotion regulation	0.81**	–				
3. Psychological resilience	0.68**	0.65**	–			
4. Resilience	0.61**	0.64**	0.90**	–		
5. Job burnout	−0.66**	−0.64**	−0.70**	−0.61**	–	
6. Emotional exhaustion	−0.60**	−0.58**	−0.65**	−0.58**	0.91**	–

**p < 0.01, all reported *p*-values survived Benjamini-Hochberg FDR correction for multiple comparisons.

Multicollinearity diagnosis showed that the VIF values for all regression models ranged between 1.23 and 1.85 (<3), indicating no severe multicollinearity, allowing for subsequent regression analysis. Using job burnout as the dependent variable, and emotional competence and psychological resilience as independent variables, regression analysis was conducted. Table 4 shows that emotional competence was significantly negatively associated with job burnout (*β* = −0.48, *p* < 0.001), and psychological resilience also significantly negatively was significantly associated with job burnout (*β* = −0.52, *p* < 0.001). Grouped regression results showed that the absolute value of the regression coefficient for the path from emotional utilization to job burnout was higher in female participants than in male participants (*β* = −0.51 for females vs. *β* = −0.38 for males). Teachers aged 31–40 had the largest coefficient on the path from psychological resilience to job burnout (*β* = −0.57). Moderated mediation analysis using PROCESS Model 14 revealed no significant gender interaction effects: the gender × emotional competence interaction on psychological resilience was not significant (*B* = 0.08, SE = 0.07, *p* = 0.26), and the gender × psychological resilience interaction on job burnout was also not significant (*B* = −0.05, SE = 0.09, *p* = 0.58). Therefore, gender does not significantly moderate the mediation pathways.

**Table 4:** Regression analysis of emotional competence, psychological resilience, and emotional utilization on job burnout and gender comparison.

Predictor variable	Model 1: total sample (Emotional competence)		Model 2: total sample (Psychological resilience)		Model 3: female sample (Emotional utilization)		Model 4: male sample (Emotional utilization)		Gender difference
	*B* (SE)	*β* (*p*)	*B* (SE)	*β* (*p*)	*B* (SE)	*β* (*p*)	*B* (SE)	*β* (*p*)	*Z* (*p*)
Emotional competence	−0.49 (0.06)	−0.48 (<0.001)	–	–	–	–	–	–	–
Psychological resilience	–	–	−0.57 (0.06)	−0.52 (<0.001)	–	–	–	–	–
Emotional utilization	–	–	–	–	−0.45 (0.07)	−0.51 (<0.001)	−0.32 (0.08)	−0.38 (<0.05)	2.13 (*p* < 0.05)
Age	–	–	–	–	0.05 (0.04)	0.06 (0.25)	0.09 (0.05)	0.11 (0.09)	–
Teaching experience	–	–	–	–	0.12 (0.05)	0.15 (< 0.05)	0.08 (0.06)	0.10 (0.18)	–
Model fit									
Adjusted R^2^	0.23	–	0.27	–	0.27	–	0.19	–	–
F	45.62**	–	52.38**	–	31.24**	–	22.78**	–	–

**p < 0.01.

All reported *p*-values were corrected for multiple comparisons using the Benjamini-Hochberg FDR method. *B*, unstandardized coefficient; SE, standard error; *β*, standardized coefficient.

**Figure 1:**
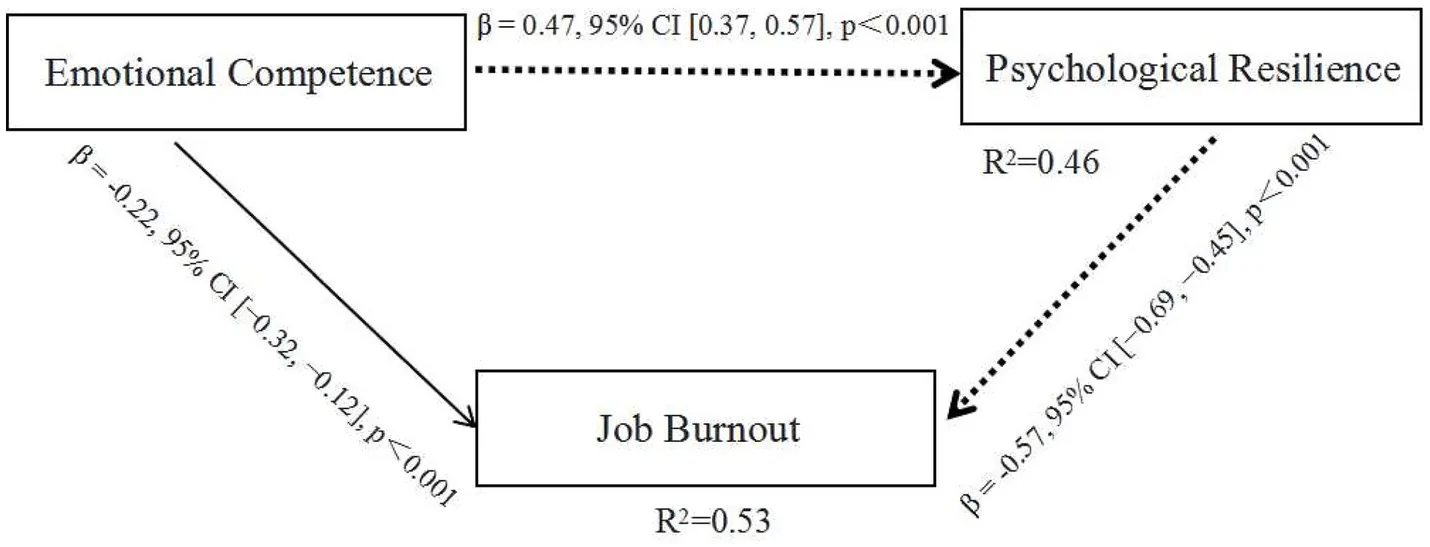
The mediating role of psychological resilience between emotional competence and job burnout. Standardized path coefficients are shown. The direct effect of emotional competence on job burnout is −0.22. The indirect effect through psychological resilience is the product of path *a* (0.47) and path *b* (−0.57).

The mediation effect test is shown in Table 5. The total association between emotional competence and job burnout was significant (*B* = −0.49, SE = 0.06, *p* < 0.001). After adding psychological resilience, the direct effect remained significant (*B* = −0.22, SE = 0.05, *p* < 0.001), but the absolute value of the coefficient decreased by 55.1%. The effect of psychological resilience on job burnout was significant (*B* = −0.57, SE = 0.06, *p* < 0.001). Results from 5,000 Bootstrap samples showed that the indirect effect was −0.27 (SE = 0.04), and the 95% confidence interval (CI) [−0.35, −0.20] did not include zero. This indicates that psychological resilience plays a partial mediating role between emotional competence and job burnout, accounting for 55.1% of the total association (indirect effect = −0.27, total effect = −0.49, proportion = |−0.27/−0.49| × 100% ≈ 55.1%). Figure 1 shows the path coefficients of emotional competence affecting job burnout through psychological resilience. The direct path coefficient from emotional competence to job burnout was −0.22, the path coefficient from emotional competence to psychological resilience was 0.47, and the path coefficient from psychological resilience to job burnout was −0.57, indicating that psychological resilience plays a partial mediating role between emotional competence and job burnout.

**Table 5:** Test results of the mediating effect of psychological resilience between emotional competence and job burnout in primary school PE teachers.

Prediction path	*β*	SE	95% CI	*p*
Total effect	−0.49	0.06	[−0.61, −0.37]	<0.001
Direct effect	−0.22	0.05	[−0.32, −0.12]	<0.001
Indirect effect	−0.27	0.04	[−0.35, −0.20]	<0.001
Emotional competence → Psychological resilience	0.47	0.05	[0.37, 0.57]	<0.001
Psychological resilience → Job burnout	−0.57	0.06	[−0.69, −0.45]	<0.001

## Discussion

This study found that emotional competence and its various dimensions significantly negatively was significantly associated with job burnout among primary school PE teachers, with the “emotion regulation” dimension having the strongest predictive power. This is consistent with findings from studies on kindergarten teachers (Zhang et al., 2025) and university teachers (Rey et al., 2016), and validates this path in the primary school physical education context. Due to the high openness, high physical interaction, and high safety risks of PE classes, teachers must regulate their emotions promptly in response to unexpected situations, such as student disputes, accidental injuries, and equipment failures, to avoid emotional exhaustion. The frequent use of deep emotional labor strategies (such as reappraising event meaning, shifting focus of attention) can directly reduce physiological arousal levels, reduce fatigue accumulation, and thus buffer emotional exhaustion and reduced personal accomplishment (Ghanizadeh and Royaei, 2015). Furthermore, teachers with high emotional competence are more adept at creating a safe and pleasant classroom atmosphere through positive emotional expression, enhancing student participation and immediate feedback, thereby strengthening the teacher’s sense of teaching efficacy and professional meaning.

Regression analysis showed that the predictive coefficient of psychological resilience for job burnout was higher than that of emotional competence. This aligns with the “resilience-resource” model proposed by Gu and Day (2007): psychological resilience reduces depersonalization tendencies by enhancing teachers’ sense of security in the face of role conflicts and external evaluation pressures; simultaneously, it alleviates reduced personal accomplishment through rapid goal adjustment and meaning reconstruction (Coffey et al., 2014). This study further found that the “Resilience” dimension had the highest correlation with burnout (*r* = −0.61). This study analyze that the trait of being able to persist and rebound from failure is particularly crucial for maintaining the professional motivation of primary school PE teachers when repeatedly facing pressures such as public lesson evaluations, competition results, and parental misunderstandings.

In this study, the total effect of emotional competence on job burnout (*β* = −0.49), the direct effect (*β* = −0.22), and the indirect effect of psychological resilience (*β* = −0.27) were all significant, fully meeting the criteria for partial mediation, thus verifying hypothesis H3. Mediation analysis indicated that psychological resilience played a partial mediating role accounting for 55.1% of the effect of emotional competence on job burnout, validating the path where emotion regulation leads to enhanced resilience, which in turn alleviates burnout. Individuals with high emotional competence first reduce negative emotional activation through cognitive reappraisal and expressive suppression, then expand their psychological resource pool (e.g., confidence, hope, social support) through the broaden-and-build function of positive emotions, thereby enhancing psychological resilience (Quoidbach et al., 2015). Subsequently, highly resilient individuals are more likely to initiate problem-solving and meaning-making strategies in the face of stressful events, further reducing the risk of job burnout (West et al., 2020). Furthermore, Bootstrap results showed that the pathway running from emotion regulation to resilience and then to burnout accounted for more than half of the total mediating association, suggesting that intervention designs could prioritize emotion regulation skills (e.g., breathing relaxation, self-talk) combined with resilience training (e.g., goal decomposition, small-step feedback).

The “psychological resilience → burnout” coefficient was largest in the 31–40 age group, possibly due to dual pressures from work and family at this life stage. Educational administrators could offer career planning and work-life balance counseling for middle-aged teachers. On a theoretical level, this study verifies the mechanism through which emotional competence indirectly affects job burnout among primary school PE teachers via psychological resilience, enriching integrated models in the field of teacher occupational health. It also refines the two key sub-dimensions of emotion regulation and resilience, providing an empirical basis for developing precise intervention indicators. On a practical level, it is recommended that educational administrative departments incorporate emotion regulation and resilience training into mandatory continuing education modules for PE teachers, and enhance group resilience through experienced teachers sharing failure and recovery experiences.

This study has certain limitations. First, this study adopted a cross-sectional design, so causal relationships cannot be inferred. Second, all data relied on self-report scales, which may be affected by common method bias. Third, the gender distribution of the sample was unbalanced, which may affect the generalizability of gender-related results. Future studies can adopt longitudinal or time-lagged designs to explore the dynamic relationships among variables. Researchers can expand the sample size and balance gender proportion to verify relevant findings. In addition, multiple mediating or moderating variables can be introduced to construct more complex models, and practical intervention programs can be developed on the basis of empirical results.

## Conclusion

This study confirms that both emotional competence and psychological resilience can alleviate job burnout among primary school PE teachers. Specifically, psychological resilience plays a partial mediating role, accounting for 55.1% of the total effect of emotional competence on job burnout. This suggests that teacher mental health should be safeguarded through emotion regulation and resilience training, with attention paid to differences among female and middle-aged groups.

## Data Availability

The raw data supporting the conclusions of this article will be made available by the authors, without undue reservation.
